# Discharge-excluded enhanced recovery after surgery adherence and hospital length of stay after laparoscopic hysterectomy for benign disease: a secondary analysis

**DOI:** 10.3389/fsurg.2026.1907048

**Published:** 2026-09-15

**Authors:** Chi Thuong Bui, Huynh Truc My Nguyen, Dinh Nguyen Pham

**Affiliations:** 1 University of Medicine and Pharmacy at Ho Chi Minh City, Ho Chi Minh City, Vietnam; 2 Nhan Dan Gia Dinh Hospital, Ho Chi Minh City, Vietnam; 3 Children’s Hospital 1, Ho Chi Minh City, Vietnam

**Keywords:** benign gynecologic disease, enhanced recovery after surgery, ERAS adherence, implementation science, laparoscopic hysterectomy, length of stay, secondary analysis, zero-truncated poisson regression

## Abstract

**Background:**

We reassessed the association between enhanced recovery after surgery (ERAS) adherence and hospital length of stay (LOS) after laparoscopic hysterectomy for benign disease after removing discharge timing from the adherence exposure to eliminate direct mathematical overlap with LOS.

**Methods:**

This retrospective secondary analysis used routine clinical audit data from 145 adults treated under an ERAS pathway at Nhan Dan Gia Dinh Hospital, Vietnam, during January 2023–June 2024. The previous 15-item audit score was retained descriptively; the primary exposure was a discharge-excluded 14-item score analyzed continuously and as an ordered completion count. Completion count was examined using proportional-odds ordinal logistic regression. LOS was modeled using zero-truncated Poisson regression after comparing Poisson and negative-binomial alternatives. Model-standardized LOS was estimated at five observed completion counts.

**Results:**

Mean discharge-excluded adherence was 81.1% ± 5.6% (11.36 ± 0.79 of 14 items), and 144/145 patients were at or above the historical 70% threshold. In the primary ordinal model, documented chronic comorbidity was associated with lower odds of higher completion category (adjusted odds ratio 0.37, 95% CI 0.18–0.76; *p* = 0.007), but this estimate attenuated when carbohydrate loading was removed from the score (adjusted odds ratio 0.72, 95% CI 0.37–1.41; *p* = 0.337). Mean LOS was 4.2 ± 1.9 days, and median LOS was 4 days (IQR 3–4). Each 10-percentage-point increase in discharge-excluded adherence was associated with lower expected LOS (adjusted incidence rate ratio 0.79, 95% CI 0.66–0.93; *p* = 0.005), equivalent to an adjusted incidence rate ratio of 0.84 per additional completed item (95% CI 0.75–0.95; *p* = 0.005). At the five observed completion counts of 9, 10, 11, 12, and 13 items, standardized expected LOS was 6.05, 5.12, 4.35, 3.71, and 3.20 days, respectively. In the simultaneous phase-specific model, preoperative and intraoperative adherence were not independently associated with LOS, whereas postoperative adherence excluding discharge was inversely associated with LOS.

**Conclusion:**

Higher discharge-excluded ERAS adherence was associated with shorter hospitalization, but the magnitude of the association was sensitive to influential observations, and the phase-specific signal was confined to postoperative adherence. Because postoperative adherence and recovery overlap in time, causal inference is not warranted.

## Introduction

Hysterectomy is a common definitive treatment for benign gynecologic disease. When vaginal hysterectomy is not feasible, laparoscopic hysterectomy offers advantages over abdominal hysterectomy, including shorter hospitalization and faster return to normal activities, although the balance of benefits and procedure-specific risks remains clinically important (1). Even after minimally invasive surgery, recovery is influenced by perioperative fasting, analgesia, fluid and catheter management, nutrition, mobilization, and discharge processes.

Enhanced recovery after surgery (ERAS) is a multidisciplinary, evidence-based approach that integrates interventions across the perioperative pathway to attenuate surgical stress and accelerate functional recovery (2). These principles are not unique to gynecology; related elements are central to contemporary ERAS guidance in colorectal and other surgical specialties (2, 3). The 2026 ERAS Society update for gynecologic oncology provides the most recent specialty-level framework for perioperative recovery, although oncology guidance is not fully interchangeable with benign hysterectomy care and procedure-specific minimally invasive gynecology guidance remains important (4, 5). In gynecologic surgery, the key implementation question is therefore not whether early feeding, opioid-sparing analgesia, mobilization, and avoidance of unnecessary tubes or drains are uniquely gynecologic, but how consistently these general recovery principles can be delivered within the workflows and clinical constraints of hysterectomy care (4, 6). Systematic reviews and implementation studies support reduced LOS and morbidity when ERAS pathways are successfully implemented (7, 8).

Protocol presence alone does not ensure protocol fidelity. Multicenter audit-and-feedback work and surveys of gynecologic practice have shown persistent variation in adherence, particularly for postoperative elements that depend on coordinated decisions by surgeons, anesthesiologists, nurses, patients, and caregivers (8, 9). Previous gynecologic studies have also related higher overall adherence to improved recovery (10, 11). A 70% adherence threshold has been used in prior gynecologic ERAS literature, including work by Pache et al., and was adopted in the original institutional audit as a pragmatic historical benchmark (12). It is not a biologically validated threshold and should not be interpreted as an intrinsic boundary between effective and ineffective care.

Descriptive results from this fixed cohort were previously published in Vietnamese, including component-level ERAS adherence, hospital LOS, and 30-day complications (13). The present study is a secondary analysis with a distinct analytical objective. The prior report did not evaluate a discharge-excluded adherence exposure, proportional-odds modeling of the corrected completion count, formal count-model comparison for LOS, model-standardized LOS at the observed corrected completion levels, or the influence, score-construction, and phase-specific sensitivity analyses reported here. The present analysis therefore addresses whether the adherence–LOS association remains after removing discharge timing from the exposure and explicitly examines the extent to which the observed association may reflect score construction, influential observations, and temporal overlap between postoperative adherence and recovery.

## Materials and methods

### Study design, setting, and reporting

This secondary analysis used a fixed retrospective dataset from the Department of Obstetrics and Gynecology, Nhan Dan Gia Dinh Hospital, a tertiary referral hospital in Ho Chi Minh City, Vietnam. The study period, 1 January 2023 through 30 June 2024, was prespecified for the original institutional audit and corresponded to a stable period of the local ERAS pathway. The exact institutional date of ERAS pathway introduction was not recorded in the locked source dataset; the analyzed interval represents a stable post-implementation period. All eligible procedures during this interval were included, and the cohort was not extended after inspection of outcomes or reviewer comments. Reporting was informed by the Strengthening the Reporting of Observational Studies in Epidemiology (STROBE) statement, the RECORD extension for routinely collected health data, and the RECOvER checklist for ERAS research (14–16).

This is a secondary analysis of a fixed cohort previously reported in Vietnamese (13). The present analysis redefines the primary adherence exposure to exclude discharge timing, analyzes adherence continuously and ordinally, applies multivariable count regression to LOS, and includes influence, score-construction, phase-specific, complication-focused, and functional-form sensitivity analyses that were not undertaken in the prior descriptive report.

### Participants and cohort assembly

Eligible participants were adults aged 18 years or older undergoing elective total or subtotal laparoscopic hysterectomy for benign pathology under the institutional ERAS pathway and classified as American Society of Anesthesiologists (ASA) physical status I–III. Exclusion criteria in the original protocol included extensive bowel adhesions requiring major adhesiolysis, concomitant surgery beyond the gynecologic procedure, conversion to open laparotomy, emergency surgery, acute pelvic inflammatory disease, and tubo-ovarian abscess.

During the study interval, 163 patients were scheduled or indicated for elective laparoscopic hysterectomy. Seven declined the planned surgical procedure and 11 procedures were converted to open laparotomy, leaving 145 completed laparoscopic hysterectomies in the analytic cohort (Figure 1). No additional exclusions were made for the present secondary analysis. During source re-audit for revision, this full screening denominator replaced the earlier 151-record/148-linked description, which did not represent the complete prespecified surgical-flow denominator; the fixed 145-patient analytic cohort was unchanged.

**Figure 1:**
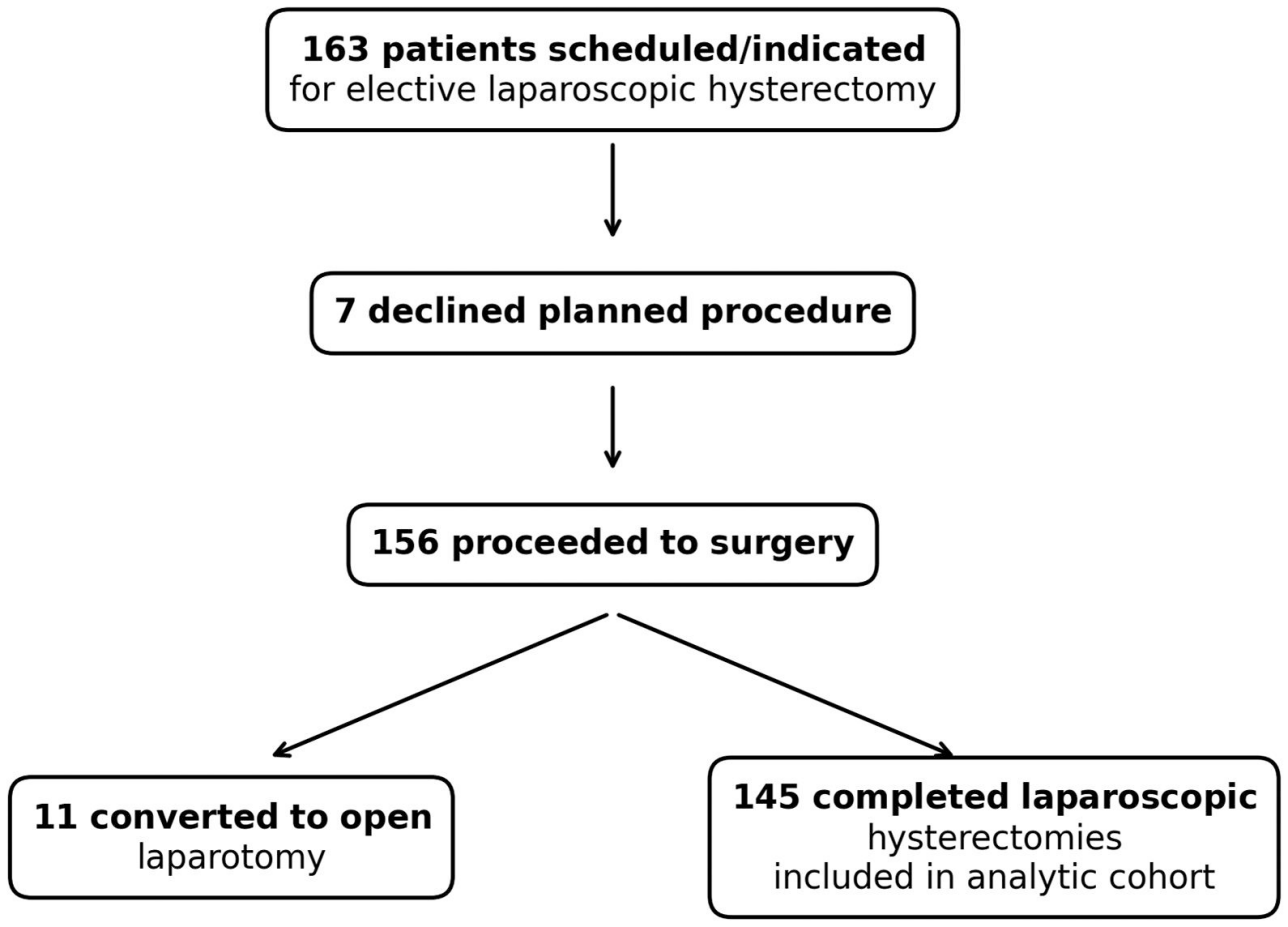
Cohort assembly. The study period was fixed *a priori* for the institutional audit. Seven patients declined the planned procedure, and 11 procedures were converted to open laparotomy; 145 completed laparoscopic hysterectomies were included.

### Study size

No *a priori* sample-size or power calculation was performed for the present secondary analysis. The analytic sample was fixed by all eligible completed laparoscopic hysterectomies in the prespecified institutional audit period. No additional patients were recruited, the study period was not extended, and no new clinical information was prospectively collected for the revision.

### ERAS pathway and phase classification

The local pathway was adapted from evidence-based perioperative principles used in gynecologic ERAS guidance (4, 6). The 2026 ERAS Society gynecologic oncology update was reviewed as the contemporary specialty framework, while recognizing that oncology recommendations are not fully interchangeable with benign laparoscopic hysterectomy care (4). The audit instrument contained 15 components: six preoperative, four intraoperative, and five postoperative items.

The preoperative items were nutritional assessment; optimization of documented chronic disease, with patients without a chronic condition considered to have met this item; avoidance of mechanical bowel preparation; administration of a locally prepared 30% maltose solution, 250 mL, approximately 2 h before surgery; intravenous cefazolin prophylaxis within 60 min before incision; and documented venous thromboembolism risk assessment using the Caprini framework. The locally documented carbohydrate formulation should not be generalized as a standard commercial ERAS drink. Pharmacologic prophylaxis concordance was not included in the reconstructed adherence item because the available risk-category and enoxaparin fields did not permit reliable patient-level reconstruction of a risk-concordant prophylaxis algorithm.

The intraoperative items were antiemetic prophylaxis with dimenhydrinate administered approximately 30 min before the end of surgery; avoidance of a nasogastric tube or removal before awakening; laparoscopic surgical access; and avoidance of routine abdominal drainage. The postoperative items were the local analgesia audit criterion, defined in the source protocol as scheduled systemic non-opioid analgesia together with a transversus abdominis plane block or wound infiltration; urinary catheter removal by postoperative day 1; oral intake within 6 h; out-of-bed mobilization within 6 h; and discharge by postoperative day 3 when clinically appropriate. Goal-directed or zero-balance fluid therapy was not included because no corresponding variable was present in the locked source dataset.

The postoperative nausea and vomiting prophylaxis item were classified as intraoperative because the institutional protocol specified administration before the end of surgery. The term “local analgesia audit criterion” is used throughout to avoid implying that patients without a regional or local technique received no analgesia. Completion was operationalized as a recorded transversus abdominis plane block or wound infiltration. Paracetamol exposure was universal and therefore did not discriminate completion of this reconstructed audit item. Eight of the 14 non-discharge items were completed in all 145 patients and contributed no between-patient variation; observed variation arose from carbohydrate loading, abdominal drainage, urinary-catheter removal, oral intake, mobilization, and the local analgesia item. Detailed operational definitions, eligibility handling, and source-variable mapping for all ERAS audit components are provided in Supplementary Table S1.

### Adherence definitions and handling of conditional items

For continuity with the previously published descriptive audit, the legacy score was calculated as the number of completed items divided by 15. The historical threshold of at least 70% was retained only for descriptive comparison with earlier gynecologic ERAS literature (12, 13). Because discharge by postoperative day 3 is directly related to LOS, it was removed from the primary exposure. The corrected score therefore included 14 non-discharge items and was expressed as both a completion counts from 0 to 14 and a percentage.

The source case-report form coded the 15 audit items as completed or not completed and did not contain a general “not applicable” category. Accordingly, the primary discharge-excluded score preserved a fixed denominator of 14 for every patient. Patients without chronic disease satisfied the chronic-disease optimization item. The VTE item was operationalized as documented Caprini risk assessment; no inference about prophylaxis concordance was made from the internally inconsistent risk-category and enoxaparin fields.

All 18 patients with diabetes were recorded as not receiving carbohydrate loading. Because the source protocol did not formally code whether diabetes represented a contraindication or a non-applicable status, these records remained non-completions in the primary fixed-denominator analysis. Three score-construction sensitivity analyses were therefore specified: carbohydrate loading was treated as not applicable for patients with diabetes; the carbohydrate item was removed from the score for all patients, creating a 13-item score; and diabetes was added as a separate covariate to the primary 14-item ordinal model. These analyses were used to evaluate whether an association between chronic comorbidity and adherence was partly induced by the scoring rule itself.

### Variables and outcomes

Variables reproducible from the source workbook included age, body mass index (BMI), ASA class, broad surgical-indication group, any documented chronic comorbidity, diabetes, prior abdominal surgery, preoperative hemoglobin, operative time, estimated blood loss, ERAS components, postoperative opioid exposure, LOS, and recorded postoperative complications. Age was derived from the surgery year and year of birth. Broad indication categories were generated using a prespecified hierarchy because the source indicator fields were not mutually exclusive: endometrial indications were assigned first, myometrial indications next, and remaining records were classified as other benign indications. Menopausal status was not systematically captured and was not inferred from age.

A formal Charlson Comorbidity Index was not available and could not be reconstructed from a complete set of diagnostic codes. Comorbidity was therefore represented by the directly recorded indicator for any chronic comorbidity. Preoperative anemia was derived from measured hemoglobin and defined as hemoglobin below 120 g/L. The source summary anemia flag disagreed with the measured-hemoglobin definition in five deidentified records; the objective hemoglobin-derived definition was used, and the discrepancy is documented in Supplementary Table S9.

The primary clinical outcome was postoperative hospital LOS in completed days, taken from the dedicated LOS field in the source dataset. During data-quality review, 16 records contained calendar dates with ambiguous day-month formatting or chronology inconsistent with the dedicated LOS field. Because the dedicated LOS field was internally consistent with the original study distribution, exact calendar dates were not used to derive LOS, and no patient was excluded based on a date-format anomaly.

Thirty-day complications were identified from component-specific complication fields and graded using the Clavien-Dindo framework (17). Five patients had a verified component-defined complication. A separate summary flag marked six patients because one record contained a positive summary flag with no positive component field; the reproducible component-defined algorithm was therefore used for the primary complication variable. Sensitivity analyses also used the six-record summary definition. Thirty-day readmission was defined by a documented readmission date in the institutional record; no readmission date was recorded. Postoperative exposure to tramadol or morphine was available as a binary indicator; opioid dose equivalents, standardized pain scores, and postoperative nausea or vomiting outcomes were not systematically captured and were not created retrospectively.

### Bias and data-quality safeguards

The revision used the locked 145-patient cohort and did not permit outcome-driven expansion of the sampling window. The analytic team had authorized access to the locked source workbook used to create the analysis dataset. No external validation study of the local audit coding or cohort-identification rules was available; operational definitions were reconciled against recorded source fields and the original institutional audit definitions before revised models were fitted.

Source cleaning was deterministic and documented. The oral-intake and mobilization variables were coded in the reverse direction from most completion fields; their polarity was corrected so that the source value indicating completion within 6 h reproduced the audit counts of 52/145 and 20/145, respectively. Data-quality safeguards also included deriving anemia from measured preoperative hemoglobin, retaining the dedicated LOS field rather than re-deriving LOS from ambiguous calendar dates, and reconciling the complication outcome from component-specific fields. Structurally unavailable variables were not created retrospectively and were excluded from analyses requiring them. The complete reconciliation of source-field anomalies and deterministic cleaning decisions is documented in Supplementary Table S9.

Potential reverse causation was reduced by excluding the discharge item from the adherence exposure and examined further through complication-focused, influence, and phase-specific sensitivity analyses. Nevertheless, postoperative milestone completion still overlaps temporally with clinical recovery, so residual reverse causation cannot be eliminated in this retrospective dataset.

### Statistical analysis

Continuous variables are reported as mean ± standard deviation and, for skewed variables, median and interquartile range. Categorical variables are reported as counts and percentages. Shapiro–Wilk tests were used only as exploratory distributional checks and were not treated as automatic decision rules.

The legacy 15-item score was summarized descriptively. The discharge-excluded 14-item score was analyzed continuously and as an ordered completion count. Because removal of the discharge item yielded 144 patients at or above the historical 70% threshold and only one below it, no inferential high-vs.-low adherence model was fitted, and no alternative cutoff was selected *post hoc*.

Factors associated with the discharge-excluded completion count were examined using proportional-odds ordinal logistic regression. Prespecified predictors available and reproducible in the locked source dataset were age per 10 years, BMI per 5 kg/m^2^, any documented chronic comorbidity, operative time per 10 min, and broad indication group. Heteroskedasticity-consistent sandwich standard errors were used, and results are reported as adjusted odds ratios for being in a higher completion category with 95% confidence intervals. Because all 18 patients with diabetes were recorded as not receiving carbohydrate loading, score-construction sensitivity analyses treated carbohydrate loading as not applicable in diabetes, removed the carbohydrate item for all patients, and added diabetes separately to the primary 14-item ordinal model.

LOS was treated as a strictly positive count outcome. Standard Poisson, negative-binomial, zero-truncated Poisson, and zero-truncated negative-binomial specifications were evaluated. The standard Poisson Pearson dispersion statistic provided no evidence of overdispersion, the negative-binomial dispersion estimate approached the Poisson boundary, and the zero-truncated negative-binomial model did not identify a stable positive dispersion parameter. The zero-truncated Poisson model had the lowest supported Akaike information criterion and was selected as the primary model. An explicit finite-sample HC1 sandwich covariance was used. Formal comparison of the candidate LOS count models is provided in Supplementary Table S6.

The primary LOS model included discharge-excluded adherence per 10 percentage points and adjusted for age, BMI, any chronic comorbidity, prior abdominal surgery, preoperative anemia, operative time, estimated blood loss, ASA physical status, and broad indication group. Results are reported as adjusted incidence rate ratios with 95% confidence intervals. Because one additional completed item corresponds to 7.14 percentage points on the 14-item scale, the adherence coefficient was also algebraically rescaled and reported as the equivalent incidence rate ratio per one additional completed item; this is the same fitted model parameter expressed on a clinically interpretable scale rather than a separately fitted model.

Model-standardized expected LOS was calculated at the five completion counts observed in the cohort: 9, 10, 11, 12, and 13 of 14 items, corresponding to 64.3%, 71.4%, 78.6%, 85.7%, and 92.9% adherence. Predictions were standardized over the observed covariate distribution. Uncertainty was estimated from 50,000 draws from the asymptotic multivariate normal distribution defined by the HC1 covariance matrix using random seed 1907048.

Sensitivity analyses excluded the five component-defined postoperative complications; excluded all six records positive on the broader complication summary flag; added either complication definition as a covariate; removed the longest stays individually and together; recalculated adherence after alternative carbohydrate-loading rules; and modeled preoperative, intraoperative, and postoperative adherence percentages simultaneously, with discharge excluded from the postoperative phase. Selected-model diagnostics included residual dispersion and exact leave-one-out refitting to quantify case-deletion influence on the adherence coefficient. Functional-form sensitivity was assessed by adding a quadratic term to the centered adherence model and repeating the comparison after deletion of the most influential lowest-adherence observation.

All variables used in the primary adherence and LOS models were complete after deterministic cleaning, with 0/145 missing observations for each model variable. Consequently, no missing-data procedure was required for the primary analyses. The variable-level distributional and missingness audit is provided in Supplementary Table S2.

Analyses were performed in Python 3.13.5 using pandas 2.2.3, NumPy 2.3.5, SciPy 1.17.0, and statsmodels 0.14.6. All tests were two-sided, with *p* < 0.05 considered statistically significant.

### Ethics and confidentiality

The study protocol was approved by the Ethics Committee in Biomedical Research of Nhan Dan Gia Dinh Hospital, Ho Chi Minh City, Vietnam (No. 01/NDGD-HDDD; 23 January 2025). The committee waived the requirement for written informed consent because this was a retrospective review of existing coded clinical records, with no prospective participant enrollment or study-specific intervention. The clinical cohort comprised patients who underwent surgery between January 2023 and June 2024; research-related review and analysis of the existing records were conducted retrospectively under the approved protocol.

## Results

### Cohort and baseline characteristics

Of 163 patients scheduled or indicated for elective laparoscopic hysterectomy during the prespecified study period, seven declined the planned procedure and 11 were converted to open laparotomy. The remaining 145 patients completed laparoscopic hysterectomy and constituted the analytic cohort (Figure 1).

Mean age was 51.7 ± 9.0 years and mean BMI was 23.7 ± 3.0 kg/m^2^. ASA classes I, II, and III accounted for 25 (17.2%), 90 (62.1%), and 30 (20.7%) patients, respectively. Sixty-six patients (45.5%) had at least one documented chronic comorbidity, 44 (30.3%) had prior abdominal surgery, and 53 (36.6%) had preoperative anemia. Mean operative time was 155.7 ± 46.4 min and median operative time was 150 min (IQR 115–180). Baseline and operative characteristics are summarized in Table 1.

**Table 1:** Baseline demographic, clinical, and operative characteristics (*N* = 145).

Characteristic	Mean ± SD or *n* (%)	Median (IQR)
Age, years	51.7 ± 9.0	50.0 (46.0–56.0)
BMI, kg/m²	23.7 ± 3.0	23.4 (21.8–25.8)
ASA I	25 (17.2)	
ASA II	90 (62.1)	
ASA III	30 (20.7)	
Endometrial indication	45 (31.0)	
Myometrial indication	72 (49.7)	
Other benign indication	28 (19.3)	
Any chronic comorbidity	66 (45.5)	
Hypertension	44 (30.3)	
Diabetes mellitus	18 (12.4)	
Chronic pulmonary disease	4 (2.8)	
Prior abdominal surgery	44 (30.3)	
Preoperative anemia, Hb <120 g/L	53 (36.6)	
Operative time, min	155.7 ± 46.4	150 (115–180)
Estimated blood loss, mL	125.4 ± 130.1	100 (50–100)

Values are mean ± SD, median (IQR), or *n* (%). ASA, American Society of Anesthesiologists; BMI, body mass index; Hb, hemoglobin; IQR, interquartile range; SD, standard deviation. Menopausal status was not systematically captured and was not inferred from age. Comorbidity categories are not mutually exclusive; 10 patients had both hypertension and diabetes mellitus. Nine patients had an additional chronic condition recorded in the free text “other condition” field.

### ERAS adherence

The legacy 15-item score averaged 11.66 ± 1.04 completed items, corresponding to 77.7% ± 6.9% adherence. Using the historical 70% threshold, 130 patients (89.7%) were at or above 70% and 15 (10.3%) were below 70%, reproducing the descriptive classification reported previously (13).

After discharge by postoperative day 3 was removed from the exposure, the discharge-excluded score averaged 11.36 ± 0.79 of 14 items, corresponding to 81.1% ± 5.6% adherence, with a range of 64.3% to 92.9%. Completion counts were 9 items in 1 patient, 10 in 14, 11 in 73, 12 in 46, and 13 in 11. Under the historical 70% threshold, 144/145 patients were at or above 70%, so binary adherence inference was not performed. All variables entering the primary adherence and LOS models were complete after deterministic cleaning, with 0/145 missing observations for every primary model variable. Definitions of the legacy and discharge-excluded scores and the observed corrected completion-count distribution are summarized in Supplementary Table S3.

Preoperative and intraoperative adherence was high for most recorded elements. Carbohydrate loading was documented in 127 patients (87.6%) and avoidance of abdominal drainage in 140 (96.6%). The principal gaps were postoperative: the local analgesia audit criterion incorporating a transversus abdominis plane block or wound infiltration was met in 7 patients (4.8%), oral intake within 6 h in 52 (35.9%), and mobilization within 6 h in 20 (13.8%). Urinary catheter removal by postoperative day 1 was documented in 141 (97.2%). Discharge by postoperative day 3, reported descriptively but excluded from the primary exposure, occurred in 44 patients (30.3%).

Adherence varied substantially across individual ERAS components, with the greatest implementation gaps observed for local analgesia, early oral intake, and early mobilization. Component-level completion rates and phase classification are presented in Table 2; detailed operational definitions, eligibility handling, and source-variable mapping are provided in Supplementary Table S1.

**Table 2:** Phase-specific adherence to the institutional ERAS audit components.

Phase	Component	Completed n/N (%)	14-item score
Preoperative	Nutritional assessment	145/145 (100.0)	Included
Preoperative	Chronic disease optimization or no disease requiring optimization	145/145 (100.0)	Included
Preoperative	30% maltose solution, 250 mL, approximately 2 h preoperatively	127/145 (87.6)	Included
Preoperative	No mechanical bowel preparation	145/145 (100.0)	Included
Preoperative	Cefazolin prophylaxis within 60 min before incision	145/145 (100.0)	Included
Preoperative	Documented VTE risk assessment using Caprini framework	145/145 (100.0)	Included
Intraoperative	PONV prophylaxis before end of surgery	145/145 (100.0)	Included
Intraoperative	No nasogastric tube or removal before awakening	145/145 (100.0)	Included
Intraoperative	No abdominal drain	140/145 (96.6)	Included
Intraoperative	Laparoscopic approach	145/145 (100.0)	Included
Postoperative	Local analgesia audit criterion: TAP block or wound infiltration	7/145 (4.8)	Included
Postoperative	Urinary catheter removal by POD1	141/145 (97.2)	Included
Postoperative	Oral intake within 6 h	52/145 (35.9)	Included
Postoperative	Out-of-bed mobilization within 6 h	20/145 (13.8)	Included
Postoperative	Discharge by POD3	44/145 (30.3)	Excluded

ERAS, enhanced recovery after surgery; POD, postoperative day; PONV, postoperative nausea and vomiting; TAP, transversus abdominis plane; VTE, venous thromboembolism. Detailed operational definitions, eligibility handling, and source-variable mapping are provided in Supplementary Table S1.

### Exploratory analysis of factors associated with discharge-excluded adherence

In the primary fixed-denominator proportional-odds model, any documented chronic comorbidity was associated with lower odds of being in a higher ERAS completion category (adjusted odds ratio 0.37, 95% CI 0.18–0.76; *p* = 0.007). This estimate was sensitive to carbohydrate-score construction. When carbohydrate loading was treated as not applicable for patients with diabetes, the comorbidity estimate attenuated to 0.49 (95% CI 0.25–0.97; *p* = 0.041). When the carbohydrate item was removed for all patients, it attenuated to 0.72 (95% CI 0.37–1.41; *p* = 0.337). When diabetes was added separately to the primary 14-item model, the comorbidity estimate was 0.64 (95% CI 0.31–1.33; *p* = 0.231). The primary comorbidity association was therefore considered score-construction-sensitive rather than a robust independent implementation finding. The complete carbohydrate-loading and diabetes score-construction sensitivity analyses are reported in Supplementary Table S5.

Operative time was not independently associated with higher completion (adjusted odds ratio 0.93 per 10 min, 95% CI 0.86–1.01; *p* = 0.100). The other-indication category had an adjusted odds ratio of 3.76 vs. endometrial indication, but indication categories required hierarchical collapse and were unstable across sensitivity models; these estimates were therefore not interpreted as robust implementation determinants. Bootstrap and threshold-specific analyses are reported in Supplementary Tables S4, S4b.

In the proportional-odds model, chronic comorbidity was associated with lower odds of being in a higher ERAS completion category, whereas the remaining covariates showed weaker or less precise associations; full adjusted estimates are presented in Table 3.

**Table 3:** Exploratory multivariable proportional-odds model for the discharge-excluded 14-item ERAS completion count.

Predictor	Adjusted OR	95% CI	*p*-value
Age, per 10 years	1.19	0.75–1.89	0.457
BMI, per 5 kg/m²	1.43	0.75–2.74	0.282
Any chronic comorbidity	0.37	0.18–0.76	0.007
Operative time, per 10 min	0.93	0.86–1.01	0.100
Myometrial vs. endometrial indication	0.76	0.35–1.64	0.481
Other vs. endometrial indication	3.76	1.28–11.05	0.016

Adjusted OR >1 indicates higher odds of being in a higher completion-count category. CI, confidence interval; ERAS, enhanced recovery after surgery; OR, odds ratio. Endometrial indications are the reference category. Robust sandwich standard errors were used. This analysis is exploratory; the chronic-comorbidity estimate is sensitive to score construction and the indication estimates are unstable across sensitivity models.

### Length of stay and safety outcomes

Mean LOS was 4.2 ± 1.9 days, median LOS was 4 days (IQR 3–4), and the range was 2–19 days. Five patients stayed 2 days, 39 stayed 3 days, 72 stayed 4 days, 16 stayed 5 days, five stayed 6 days, five stayed 7 days, and one patient each stayed 11, 14, and 19 days.

After multivariable adjustment, each 10-percentage-point increase in discharge-excluded adherence was associated with lower expected LOS (adjusted incidence rate ratio 0.79, 95% CI 0.66–0.93; *p* = 0.005). Expressed on the completion-count scale, the same model coefficient corresponds to an adjusted incidence rate ratio of 0.84 per one additional completed ERAS item (95% CI 0.75–0.95; *p* = 0.005). Prior abdominal surgery was also associated with longer expected LOS (adjusted incidence rate ratio 1.28, 95% CI 1.09–1.49; *p* = 0.002). Full adjusted estimates from the primary multivariable zero-truncated Poisson model are presented in Table 4.

**Table 4:** Multivariable zero-truncated poisson regression for hospital length of stay.

Predictor	Adjusted IRR	95% CI	*p*-value
Discharge-excluded ERAS adherence, per 10 percentage points	0.79	0.66–0.93	0.005
Equivalent expression per 1 additional completed item	0.84	0.75–0.95	0.005
Age, per 10 years	0.99	0.91–1.08	0.782
BMI, per 5 kg/m²	0.98	0.86–1.11	0.739
Any chronic comorbidity	1.10	0.95–1.26	0.207
Prior abdominal surgery	1.28	1.09–1.49	0.002
Preoperative anemia, Hb <120 g/L	0.92	0.76–1.11	0.369
Operative time, per 10 min	1.01	1.00–1.03	0.110
Estimated blood loss, per 100 mL	1.00	0.96–1.04	0.969
ASA II vs. I	1.00	0.89–1.12	0.967
ASA III vs. I	1.02	0.85–1.23	0.831
Myometrial vs. endometrial indication	1.09	0.94–1.26	0.281
Other vs. endometrial indication	1.01	0.87–1.17	0.928

CI, confidence interval; ERAS, enhanced recovery after surgery; IRR, incidence rate ratio. The model used an explicit finite-sample HC1 sandwich covariance. The per-item row is an algebraic rescaling of the same adherence coefficient and is not a separately fitted parameter. ASA I and endometrial indications are reference categories.

Standardized expected LOS was estimated only at completion counts observed in the cohort. Expected LOS was 6.05 days (95% CI 4.57–8.21) at 9 of 14 completed items, 5.12 days (95% CI 4.35–6.17) at 10 items, 4.35 days (95% CI 4.07–4.73) at 11 items, 3.71 days (95% CI 3.44–4.08) at 12 items, and 3.20 days (95% CI 2.74–3.84) at 13 items. These are model-standardized predictions evaluated at observed exposure support values, not raw within-score means. The lowest completion count was represented by one patient, and its prediction should therefore be interpreted cautiously. These model-standardized estimates are displayed in Figure 2 and tabulated with observed group sizes and 95% confidence intervals in Supplementary Table S10.

**Figure 2:**
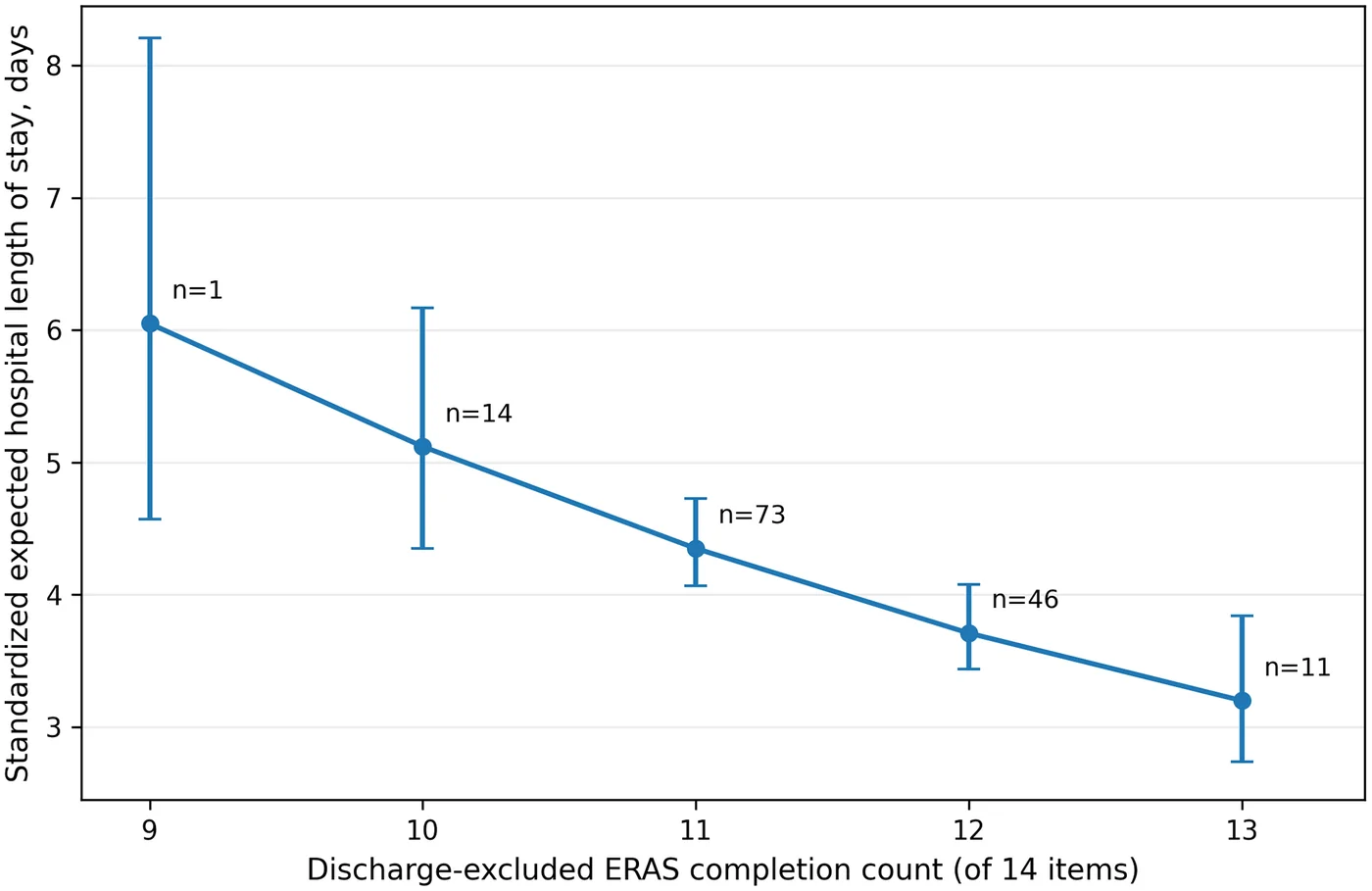
Model-standardized expected hospital LOS at the five discharge-excluded ERAS completion counts observed in the cohort. Error bars show 95% simulation intervals from 50,000 coefficient draws using the explicit HC1 covariance matrix. The *n* labels indicate the number of patients observed at each completion count.

Five of 145 patients (3.4%) had a verified 30-day postoperative complication based on component-specific fields: three localized peritonitis events, one postoperative bleeding event, and one surgical-site infection. No cardiopulmonary complication or death was recorded. No 30-day readmission date was documented in the institutional dataset. Fifty-eight patients (40.0%) received at least one postoperative opioid recorded as tramadol or morphine; 56 (38.6%) received tramadol and five (3.4%) received morphine, with three patients receiving both. Dose-equivalent opioid consumption, standardized pain scores, and postoperative nausea or vomiting rates were not available.

### Sensitivity analyses and phase-specific adherence

The inverse association between discharge-excluded adherence and LOS persisted across the prespecified sensitivity analyses, but its magnitude was not invariant. Excluding the five component-defined complications yielded an adjusted IRR of 0.86 per 10 percentage points (95% CI 0.79–0.95; *p* = 0.002), and excluding all six records positive on the broader complication summary flag yielded 0.86 (95% CI 0.78–0.95; *p* = 0.002). Adding the five-case complication indicator yielded 0.83 (95% CI 0.76–0.91; *p* < 0.001), and adding the six-record summary indicator yielded 0.81 (95% CI 0.73–0.89; *p* < 0.001). Excluding LOS values of 19, 14, or 11 days individually yielded adjusted IRRs of 0.86, 0.77, and 0.80, respectively; excluding all three longest stays together yielded 0.87 (95% CI 0.79–0.96; *p* = 0.004). Alternative carbohydrate-score constructions yielded adjusted IRRs of 0.78 when carbohydrate loading was treated as not applicable in diabetes and 0.80 when the item was removed from the score. The complete set of prespecified LOS sensitivity analyses is reported in Supplementary Table S7.

Selected-model diagnostics did not indicate residual overdispersion: Pearson residual dispersion was 0.52 and likelihood-based deviance divided by residual degrees of freedom was 0.46. Exact leave-one-out refitting identified study ID 6, the only patient with 9 of 14 completed items (64.3% adherence) and the longest LOS of 19 days, as the dominant case for the adherence coefficient, with an absolute deletion DFBETA of 1.09. Deleting this observation changed the adjusted incidence rate ratio from 0.79 to 0.86 per 10 percentage points (95% CI 0.78–0.95; *p* = 0.004). These results show that the inverse direction was stable, whereas the precise magnitude was materially influenced by the sparse lower-adherence tail. Residual and exact leave-one-out influence diagnostics are reported in Supplementary Table S6b.

Functional-form analysis also depended on the same observation. In the full cohort, adding a quadratic adherence term reduced AIC from 548.88 to 545.14 and the quadratic coefficient was 0.201 (95% CI 0.039–0.363; *p* = 0.015). After exclusion of study ID 6, the quadratic coefficient was 0.008 (95% CI −0.128 to 0.144; *p* = 0.903), while the linear model retained an inverse association and had lower AIC than the quadratic model (532.51 vs. 534.51). The direction of association was therefore more stable than its precise functional form. Full linear and quadratic model comparisons are provided in Supplementary Table S11.

In the simultaneous phase-specific model, preoperative adherence was not independently associated with LOS (adjusted incidence rate ratio 0.91 per 10 percentage points, 95% CI 0.75–1.10; *p* = 0.328), and neither was intraoperative adherence (adjusted incidence rate ratio 0.96, 95% CI 0.85–1.09; *p* = 0.537). Higher postoperative adherence excluding discharge was associated with shorter LOS (adjusted incidence rate ratio 0.93, 95% CI 0.89–0.97; *p* = 0.001). Because postoperative adherence was measured during the same recovery interval in which LOS accrued, the postoperative phase-specific association is particularly susceptible to reverse causation and is interpreted as a sensitivity finding rather than evidence of a causal postoperative treatment effect. The simultaneous phase-specific estimates are also reported in Supplementary Table S8.

## Discussion

After discharge by postoperative day 3 was removed from the adherence exposure, higher discharge-excluded ERAS adherence remained associated with shorter hospitalization after laparoscopic hysterectomy for benign disease. Three features are central to interpretation. First, the exposure excludes the discharge audit item, so no component of the primary score is mathematically coupled to LOS. Second, no high-vs.-low adherence comparison is made because 144 of 145 patients were at or above the historical 70% threshold. Third, the LOS model was selected after explicit comparison of standard and zero-truncated count families rather than assumed *a priori*.

The component profile highlights an implementation problem rather than a failure of ERAS principles themselves. Many preoperative and intraoperative elements were embedded in standardized workflows and were completed in nearly all patients, whereas postoperative feeding, mobilization, and the local analgesia criterion were less consistently documented. This pattern is consistent with gynecologic implementation literature showing that ward-based rehabilitation can be more difficult to sustain than protocolized preoperative or operating-room processes (8, 9). The 2026 ERAS Society update reinforces coordinated perioperative recovery processes in gynecologic oncology, while AAGL guidance provides procedure-specific context for minimally invasive gynecologic surgery (4, 5). The contribution of the present study is therefore the identification of where a general recovery pathway loses fidelity in routine minimally invasive hysterectomy practice.

The low 4.8% completion rate for the local analgesia audit criterion requires careful interpretation. The reconstructed item identified use of a transversus abdominis plane block or wound infiltration under the source audit definition and should not be interpreted as absence of systemic postoperative analgesia in the remaining patients. Paracetamol exposure was universal, and 40.0% of patients had a recorded postoperative exposure to tramadol or morphine, but the dataset did not contain dose equivalents or standardized pain scores. Contemporary minimally invasive gynecologic ERAS guidance emphasizes multimodal analgesia and limitation of opioid exposure, yet the present dataset cannot separate systemic multimodal therapy, regional techniques, opioid dose, pain intensity, sedation, and nausea into distinct quantitative domains (4, 5).

The other major postoperative gaps also require individualized interpretation. Earlier oral intake can support return toward normal gastrointestinal and metabolic function, while nausea, uncontrolled pain, reduced consciousness, or aspiration concern may justify delay in selected patients. Early mobilization can reduce immobilization-related deconditioning, but hypotension, dizziness, pain, bleeding concern, or immediate postoperative instability may appropriately postpone ambulation. For quality improvement, time-stamped milestone documentation should distinguish true non-completion from a documented contraindication or “not applicable” status. Nurse-led milestone documentation, escalation pathways, and multidisciplinary audit may improve measurement fidelity, but the present observational analysis does not establish the effectiveness of any specific implementation intervention. Digital patient-facing tools and structured surgical safety checklists have also been developed to support ERAS compliance and implementation (20, 21).

The exploratory ordinal analysis illustrates why score construction must be interpreted cautiously. All 18 patients with diabetes were members of the chronic-comorbidity category and were also classified as not completing carbohydrate loading. The fixed-denominator definition therefore creates a mechanical pathway by which diabetes can lower the adherence score irrespective of the patient's broader pathway implementation performance, introducing potential classification and score-construction bias into the comorbidity–adherence association. Consistent with this mechanism, the comorbidity adjusted odds ratio attenuated from 0.37 (95% CI 0.18–0.76) in the primary 14-item model to 0.72 (95% CI 0.37–1.41) when the carbohydrate-loading item was removed for all patients. This attenuation indicates that part of the primary comorbidity signal is attributable to how diabetes was coded within the adherence denominator; the coefficient should therefore not be interpreted as a robust independent determinant of implementation.

The phase-specific analysis materially informs the reverse-causation question. Preoperative adherence, determined before postoperative recovery begins, was not independently associated with LOS, and intraoperative adherence was also null. The inverse signal was confined to postoperative adherence. This pattern does not prove reverse causation, but it is consistent with the possibility that early postoperative clinical trajectory influences both milestone completion and subsequent hospitalization. Pain, nausea, hemodynamic instability, complications, and delayed functional recovery can reduce completion of feeding, mobilization, catheter, or analgesia milestones while simultaneously prolonging LOS. The postoperative phase-specific association should therefore be interpreted as a marker of pathway execution and recovery progression rather than evidence that increasing a specific postoperative item will necessarily shorten hospitalization.

The corrected point estimate was farther from the null than the estimate reported in the earlier analysis: the adjusted incidence rate ratio changed from 0.91 to 0.79 rather than attenuating after discharge timing was removed. This direction of change should not be interpreted as evidence that removing mathematical overlap uncovered a stronger causal effect. The revised exposure, model specification, and influence structure differ from the earlier analysis, and the lower end of the corrected adherence distribution is sparse. Exact leave-one-out analysis identified study ID 6, with 64.3% adherence and a 19-day stay, as the dominant observation for the adherence coefficient; the absolute deletion DFBETA was 1.09. Removing this observation attenuated the adjusted incidence rate ratio to 0.86 (95% CI 0.78–0.95; *p* = 0.004), closer to the earlier 0.91 estimate. The inverse direction is reproducible across sensitivity analyses, whereas the magnitude is sensitive to influential observations and should not be treated as a precise biological dose-response.

The corrected score takes only five observed values, so standardized LOS summaries should respect that support. Reporting model-standardized expected LOS at 9, 10, 11, 12, and 13 completed items avoids implying that arbitrary percentage points between those values were directly observed. Expressing the primary coefficient per one additional completed item also improves clinical interpretability: each additional item was associated with an estimated 16% lower expected LOS rate in the fitted model, although this is an associational summary and does not establish that adding one specific component would produce that reduction.

Prior abdominal surgery was independently associated with longer expected LOS in the primary count model. This finding may reflect greater operative complexity or recovery burden, but the present dataset was not designed to establish a mechanism. Contemporary reports show substantial variation in LOS across institutions and care models; a 2026 retrospective study of benign laparoscopic hysterectomy and a 2024 systematic review both support the broader relevance of ERAS to hospitalization while illustrating that institutional context matters (18, 19). Local discharge culture, bed-management norms, reimbursement processes, family readiness, transportation, and post-discharge support can influence LOS independently of physiological recovery.

This study has several limitations. It is a single-center retrospective secondary analysis of a fixed 145-patient cohort, limiting external generalizability and causal inference, and no *a priori* sample-size calculation was performed because the sample was fixed by the prespecified audit period. The source audit instrument used a fixed binary denominator and did not systematically distinguish contraindicated from failed items, most notably for carbohydrate loading in diabetes. Eight of the 14 scored items were completed in all patients, so observed variation derives from a small number of components. The VTE fields allowed reproducible assessment of documented Caprini risk evaluation but not reliable reconstruction of risk-concordant pharmacologic prophylaxis. Menopausal status, frailty, standardized opioid dose, validated pain scores, and postoperative nausea or vomiting outcomes were not systematically recorded. Several postoperative adherence components were measured during the same period in which LOS accrued, so reverse causation remains possible. The proportional-odds model included sparse extreme completion categories, and sensitivity analyses showed variation in magnitude and precision. The complication count was low and one summary flag was discordant with component-specific fields, although analyses using both definitions were directionally concordant. The magnitude of the adherence–LOS association was not fully invariant, and the single lowest-adherence patient with the longest stay materially influenced both the coefficient and apparent curvature. Thus, the direction of association is more robust than either its precise size or its exact functional form.

## Conclusion

In this secondary analysis of 145 patients undergoing laparoscopic hysterectomy for benign disease, higher discharge-excluded ERAS adherence was associated with shorter hospital LOS. The equivalent model estimate per one additional completed item was an adjusted incidence rate ratio of 0.84, but the magnitude of the association was influenced by the sparse lower-adherence tail. Preoperative and intraoperative adherence were not independently associated with LOS, whereas the phase-specific signal was confined to postoperative adherence, where temporal overlap with recovery makes reverse causation plausible. The chronic-comorbidity association with lower completion was sensitive to carbohydrate-score construction. These findings should therefore be interpreted as associational and hypothesis-generating and support prospective, time-resolved evaluation of postoperative pathway implementation rather than causal claims about adherence itself.

## Data Availability

The raw data supporting the conclusions of this article will be made available by the authors, without undue reservation.

## References

[1] Pickett CM Seeratan DD Mol BWJ Nieboer TE Johnson N Bonestroo T. Surgical approach to hysterectomy for benign gynaecological disease. Cochrane Database Syst Rev. (2023) 8:CD003677. 10.1002/14651858.CD003677.pub6 37642285 PMC10464658

[2] Grant MC Engelman DT. Enhanced recovery after surgery: overarching themes of the ERAS society guidelines and consensus statements for adult specialty surgery. Perioper Med. (2025) 14:120. 10.1186/s13741-025-00590-0 PMC12577217 41168801

[3] Irani JL Hedrick TL Miller TE Lee L Steinhagen E Shogan BD. Clinical practice guidelines for enhanced recovery after colon and rectal surgery from the American society of colon and rectal surgeons and society of American gastrointestinal and endoscopic surgeons. Surg Endosc. (2023) 37:5–30. 10.1007/s00464-022-09758-x 36515747 PMC9839829

[4] Nelson G Altman AD Metcalfe A Fotopoulou C Taylor J Glaser G. Enhanced recovery after surgery (ERAS®) society guidelines for gynecologic oncology: 2026 update. Gynecol Oncol. (2026) 210:153–73. 10.1016/j.ygyno.2026.05.022 42247726

[5] Stone R Carey E Fader AN Fitzgerald J Hammons L Nensi A. Enhanced recovery and surgical optimization protocol for minimally invasive gynecologic surgery: an AAGL white paper. J Minim Invasive Gynecol. (2021) 28:179–203. 10.1016/j.jmig.2020.08.006 32827721

[6] Nelson G Bakkum-Gamez J Kalogera E Glaser G Altman A Meyer LA. Guidelines for perioperative care in gynecologic/oncology: enhanced recovery after surgery society recommendations—2019 update. Int J Gynecol Cancer. (2019) 29:651–68. 10.1136/ijgc-2019-000356 30877144

[7] Bisch SP Jago CA Kalogera E Ganshorn H Meyer LA Ramirez PT. Outcomes of enhanced recovery after surgery in gynecologic oncology: a systematic review and meta-analysis. Gynecol Oncol. (2021) 161:46–55. 10.1016/j.ygyno.2020.12.035 33388155

[8] Piovano E Puppo A Camanni M Castiglione A Delpiano EM Giacometti L. Implementing enhanced recovery after surgery for hysterectomy in a hospital network with audit and feedback: a stepped-wedge cluster randomised trial. BJOG. (2024) 131:1207–17. 10.1111/1471-0528.17797 38404145

[9] Gómez-Hidalgo NR Pletnev A Razumova Z Bizzarri N Selcuk I Theofanakis C. European Enhanced recovery after surgery gynecologic oncology survey: status of ERAS protocol implementation across Europe. Int J Gynaecol Obstet. (2023) 160:306–12. 10.1002/ijgo.14386 35929452

[10] Shen Y Lv F Min S Wu G Jin J Gong Y. Impact of enhanced recovery after surgery protocol compliance on patients’ outcome in benign hysterectomy and establishment of a predictive nomogram model. BMC Anesthesiol. (2021) 21:289. 10.1186/s12871-021-01509-0 34809583 PMC8607678

[11] Hayek J Zorrilla-Vaca A Meyer LA Mena G Lasala J Iniesta MD. Patient outcomes and adherence to an enhanced recovery pathway for open gynecologic surgery: a 6-year single-center experience. Int J Gynecol Cancer. (2022) 32:1443–9. 10.1136/ijgc-2022-003840 36202425

[12] Pache B Jurt J Grass F Hübner M Demartines N Mathevet P. Compliance with enhanced recovery after surgery program in gynecology: are all items of equal importance? Int J Gynecol Cancer. (2019) 29:810–5. 10.1136/ijgc-2019-000268 30898937

[13] Nguyen HTM Bui CT Nguyen PHH Trinh TXV. Survey on compliance with the enhanced recovery after surgery (ERAS) protocol in patients undergoing laparoscopic hysterectomy at Nhan Dan Gia Dinh Hospital. Vietnam Med J. (2025) 554(1):275–8. 10.51298/vmj.v554i1.15608

[14] Vandenbroucke JP Von Elm E Altman DG Gøtzsche PC Mulrow CD Pocock SJ. Strengthening the reporting of observational studies in epidemiology (STROBE): explanation and elaboration. Int J Surg. (2014) 12:1500–24. 10.1016/j.ijsu.2014.07.014 25046751

[15] Benchimol EI Smeeth L Guttmann A Harron K Moher D Petersen I. The REporting of studies conducted using observational routinely-collected health data (RECORD) statement. PLoS Med. (2015) 12:e1001885. 10.1371/journal.pmed.1001885 26440803 PMC4595218

[16] Elias KM Stone AB McGinigle K Tankou JI Scott MJ Fawcett WJ. The reporting on ERAS compliance, outcomes, and elements research (RECOvER) checklist: a joint statement by the ERAS and ERAS USA societies. World J Surg. (2019) 43:1–8. 10.1007/s00268-018-4753-0 30116862 PMC6313353

[17] Dindo D Demartines N Clavien PA. Classification of surgical complications: a new proposal with evaluation in a cohort of 6336 patients and results of a survey. Ann Surg. (2004) 240:205–13. 10.1097/01.sla.0000133083.54934.ae 15273542 PMC1360123

[18] Liu Y Fang Y Zhao X. Effect of enhanced recovery after surgery protocol for laparoscopic hysterectomy in benign indications: a retrospective cohort study. Front Med. (2026) 13:1876613. 10.3389/fmed.2026.1876613 PMC13357842 42445157

[19] Nian J Li Z Chen P Ye P Liu C. Enhanced recovery after surgery versus conventional postoperative care in patients undergoing hysterectomy: a systematic review and meta-analysis. Arch Gynecol Obstet. (2024) 310:515–24. 10.1007/s00404-024-07475-5 38836927

[20] Beesoon S Drobot A Smokeyday M Ali A-B Collins Z Reynolds C. Patient and provider experiences with a digital app to improve compliance with enhanced recovery after surgery protocols: mixed methods evaluation of a Canadian experience. JMIR Form Res. (2023) 7:e49277. 10.2196/49277 38100170 PMC10757223

[21] Pilkington M Nelson G Cauley C Holder K Ljungqvist O Molina G. Development of an enhanced recovery after surgery surgical safety checklist through a modified delphi process. JAMA Netw Open. (2023) 6:e2248460. 10.1001/jamanetworkopen.2022.48460 36753283

