# Supplementary material for: Discharge-excluded enhanced recovery after surgery adherence and hospital length of stay after laparoscopic hysterectomy for benign disease: a secondary analysis
Source: Front Surg. 2026 Sep 15;13:1907048. doi: 10.3389/fsurg.2026.1907048 (PMC13621892; doi:10.3389/fsurg.2026.1907048)
Supplement: Supplementary file 1 [file Table1.docx]

**Supplementary Material**

**Supplementary Tables S1–S11**

## Supplementary Table S1. Operational data dictionary for ERAS audit components

| **Phase** | **Component** | **Source variable(s)** | **Operational definition** | **Eligibility handling** | **Analytic role** |
| --- | --- | --- | --- | --- | --- |
| Preoperative | Nutritional assessment | C25 | Completed if documented as performed | All patients | 14-item score |
| Preoperative | Chronic disease optimization | B7 + C27 | Completed if no chronic disease or documented optimization | Conditional logic embedded | 14-item score |
| Preoperative | Carbohydrate loading | C24 | Local 30% maltose, 250 mL, approximately 2 h before surgery | Fixed denominator in primary analysis; diabetes N/A in sensitivity; removed entirely in another sensitivity | 14-item score |
| Preoperative | No mechanical bowel preparation | C23 | Completed when mechanical bowel preparation absent | All patients | 14-item score |
| Preoperative | Antibiotic prophylaxis | C26 | Cefazolin within 60 min before incision | All patients | 14-item score |
| Preoperative | Documented VTE risk assessment | C28 + C29 | Caprini assessment documented; C29 accepted when explicit category present despite C28=0 | All patients | 14-item score |
| Intraoperative | PONV prophylaxis | D1 | Dimenhydrinate approximately 30 min before end of surgery | All patients | 14-item score |
| Intraoperative | Nasogastric tube avoidance | D2 + D3 | No tube or removed before awakening | All patients | 14-item score |
| Intraoperative | Laparoscopic approach | Cohort definition | Procedure completed laparoscopically | All analyzed patients | 14-item score |
| Intraoperative | No abdominal drain | D15 | No routine abdominal drain | All patients | 14-item score |
| Postoperative | Local analgesia audit criterion | D5 + D6 | Completion operationalized as D5=1 (TAP block) or D6=1 (wound infiltration). Paracetamol exposure was universal and did not discriminate this audit item. | All patients: systemic analgesic exposures reported separately where available | 14-item score |
| Postoperative | Urinary catheter removed by POD1 | E23 | Removal on/before POD1 | All patients | 14-item score |
| Postoperative | Oral intake within 6 h | E14 | Oral intake within 6 h | All patients | 14-item score |
| Postoperative | Mobilization within 6 h | E24 | Out of bed within 6 h | All patients | 14-item score |
| Postoperative | Discharge by POD3 | F2 | LOS <=3 completed days | All patients | Legacy 15-item score only; excluded from primary exposure |

POD, postoperative day; PONV, postoperative nausea and vomiting; TAP, transversus abdominis plane; VTE, venous thromboembolism. The VTE adherence item is narrowed relative to the source protocol definition and is restricted to documented Caprini risk assessment; pharmacologic prophylaxis concordance was not inferred from the internally inconsistent risk-category and enoxaparin fields. In the source workbook the oral-intake (E14) and mobilization (E24) fields are coded with 0 indicating completion within 6 hours; the completion counts reported here (52/145 and 20/145) reproduce the component rates of the original institutional audit. Laparoscopic access is cohort-defining and was completed in all analyzed patients.

## Supplementary Table S2. Distributional and missingness audit

| **Variable** | **Missing n/N (%)** | **Shapiro-Wilk W** | **p-value** |
| --- | --- | --- | --- |
| Age, years | 0/145 (0.0%) | 0.950 | 0.0000425 |
| BMI, kg/m² | 0/145 (0.0%) | 0.981 | 0.045 |
| Preoperative hemoglobin, g/L | 0/145 (0.0%) | 0.879 | 1.69 × 10⁻⁹ |
| Operative time, min | 0/145 (0.0%) | 0.954 | 0.0000935 |
| Estimated blood loss, mL | 0/145 (0.0%) | 0.572 | 8.45 × 10⁻¹⁹ |
| Hospital LOS, days | 0/145 (0.0%) | 0.546 | 2.49 × 10⁻¹⁹ |
| Discharge-excluded ERAS adherence, % | 0/145 (0.0%) | 0.857 | 1.56 × 10⁻¹⁰ |
| Any chronic comorbidity | 0/145 (0.0%) | Not applicable | Not applicable |
| Prior abdominal surgery | 0/145 (0.0%) | Not applicable | Not applicable |
| Preoperative anemia, Hb <120 g/L | 0/145 (0.0%) | Not applicable | Not applicable |
| ASA physical status | 0/145 (0.0%) | Not applicable | Not applicable |
| Broad indication group | 0/145 (0.0%) | Not applicable | Not applicable |
| Diabetes mellitus | 0/145 (0.0%) | Not applicable | Not applicable |

Shapiro-Wilk testing was exploratory and was not used as an automatic decision rule. All variables used in the primary adherence and LOS models were complete after deterministic cleaning, with 0/145 missing observations for each model variable. Consequently, no missing-data procedure was required for the primary analyses.

## Supplementary Table S3. ERAS score definitions and observed completion-count distribution

| **Definition** | **Denominator** | **Completed items, mean ± SD** | **Adherence %, mean ± SD** | **Range, %** | **≥70%** | **<70%** |
| --- | --- | --- | --- | --- | --- | --- |
| Legacy score including discharge | 15 | 11.66 ± 1.04 | 77.7 ± 6.9 | 60.0–93.3 | 130/145 | 15/145 |
| Discharge-excluded score | 14 | 11.36 ± 0.79 | 81.1 ± 5.6 | 64.3–92.9 | 144/145 | 1/145 |

Observed discharge-excluded 14-item completion counts:

| **Completed items** | **N** | **Percent** |
| --- | --- | --- |
| 9 | 1 | 0.7 |
| 10 | 14 | 9.7 |
| 11 | 73 | 50.3 |
| 12 | 46 | 31.7 |
| 13 | 11 | 7.6 |

The historical 70% threshold cannot support a binary inferential model after discharge is removed because only one patient remains below 70%. No alternative data-driven cutoff was introduced. Eight of the 14 non-discharge items were completed in all 145 patients and therefore contributed no between-patient variation; observed variation in the score arose from the carbohydrate-loading, abdominal-drain, urinary-catheter, oral-intake, mobilization, and local-analgesia items.

## Supplementary Table S4. Nonparametric bootstrap stability of the ordinal adherence model

| **Predictor** | **Successful replicates** | **OR 2.5th percentile** | **Median OR** | **OR 97.5th percentile** |
| --- | --- | --- | --- | --- |
| Age, per 10 years | 1000 | 0.75 | 1.22 | 1.99 |
| BMI, per 5 kg/m2 | 1000 | 0.77 | 1.45 | 2.85 |
| Any chronic comorbidity | 1000 | 0.17 | 0.36 | 0.74 |
| Operative time, per 10 minutes | 1000 | 0.85 | 0.93 | 1.01 |
| Myometrial vs endometrial indication | 1000 | 0.33 | 0.76 | 1.72 |
| Other vs endometrial indication | 1000 | 1.38 | 4.09 | 12.60 |

**Supplementary Table S4b. Sensitivity assessment of the proportional-odds structure**

| **Analysis** | **Predictor** | **Adjusted OR** | **95% CI** | **p-value** | **Threshold n (≥ / <)** |
| --- | --- | --- | --- | --- | --- |
| Collapsed 9/10 category ordinal model | Age, per 10 years | 1.19 | 0.75–1.89 | 0.465 | — |
| Collapsed 9/10 category ordinal model | BMI, per 5 kg/m2 | 1.38 | 0.74–2.58 | 0.306 | — |
| Collapsed 9/10 category ordinal model | Any chronic comorbidity | 0.38 | 0.19–0.76 | 0.006 | — |
| Collapsed 9/10 category ordinal model | Operative time, per 10 minutes | 0.94 | 0.86–1.01 | 0.106 | — |
| Collapsed 9/10 category ordinal model | Myometrial vs endometrial indication | 0.76 | 0.35–1.67 | 0.501 | — |
| Collapsed 9/10 category ordinal model | Other vs endometrial indication | 3.73 | 1.27–10.91 | 0.016 | — |
| Binary threshold: score >= 11 | Age, per 10 years | 1.03 | 0.45–2.36 | 0.953 | 130 / 15 |
| Binary threshold: score >= 11 | BMI, per 5 kg/m2 | 1.32 | 0.40–4.36 | 0.654 | 130 / 15 |
| Binary threshold: score >= 11 | Any chronic comorbidity | 0.15 | 0.04–0.58 | 0.007 | 130 / 15 |
| Binary threshold: score >= 11 | Operative time, per 10 minutes | 0.88 | 0.78–0.99 | 0.038 | 130 / 15 |
| Binary threshold: score >= 11 | Myometrial vs endometrial indication | 1.23 | 0.37–4.15 | 0.734 | 130 / 15 |
| Binary threshold: score >= 11 | Other vs endometrial indication | 4.73 | 0.53–42.20 | 0.164 | 130 / 15 |
| Binary threshold: score >= 12 | Age, per 10 years | 1.14 | 0.73–1.77 | 0.571 | 57 / 88 |
| Binary threshold: score >= 12 | BMI, per 5 kg/m2 | 1.26 | 0.66–2.42 | 0.485 | 57 / 88 |
| Binary threshold: score >= 12 | Any chronic comorbidity | 0.51 | 0.23–1.12 | 0.095 | 57 / 88 |
| Binary threshold: score >= 12 | Operative time, per 10 minutes | 0.95 | 0.87–1.04 | 0.239 | 57 / 88 |
| Binary threshold: score >= 12 | Myometrial vs endometrial indication | 0.59 | 0.23–1.51 | 0.271 | 57 / 88 |
| Binary threshold: score >= 12 | Other vs endometrial indication | 2.74 | 0.92–8.22 | 0.071 | 57 / 88 |
| Binary threshold: score >= 13 | Age, per 10 years | 1.68 | 0.70–4.02 | 0.242 | 11 / 134 |
| Binary threshold: score >= 13 | BMI, per 5 kg/m2 | 1.53 | 0.62–3.80 | 0.359 | 11 / 134 |
| Binary threshold: score >= 13 | Any chronic comorbidity | 0.36 | 0.08–1.56 | 0.171 | 11 / 134 |
| Binary threshold: score >= 13 | Operative time, per 10 minutes | 0.99 | 0.89–1.11 | 0.882 | 11 / 134 |
| Binary threshold: score >= 13 | Myometrial vs endometrial indication | 1.41 | 0.26–7.56 | 0.688 | 11 / 134 |
| Binary threshold: score >= 13 | Other vs endometrial indication | 8.64 | 1.39–53.58 | 0.021 | 11 / 134 |

Because the lowest completion category contained one patient, scores of 9 and 10 were combined in a four-level ordinal sensitivity model. Adjusted binary logistic models were also fitted at thresholds of at least 11, 12, and 13 completed items. A formal Brant test was not relied upon because sparse extreme categories can make threshold-specific inference unstable. The comorbidity coefficient remained below 1 at all thresholds, but effect magnitude and precision varied; these analyses therefore support cautious interpretation rather than a definitive claim that proportional odds was established.

A total of 1,000 patient-level bootstrap replicates were generated with random seed 1907048. Bootstrap distributions were used as a model-stability sensitivity analysis for the proportional-odds model.

## Supplementary Table S5. Sensitivity of the comorbidity–adherence association to carbohydrate-score construction

| **Analysis** | **Comorbidity aOR** | **95% CI** | **p-value** |
| --- | --- | --- | --- |
| Primary 14-item fixed denominator | 0.37 | 0.18–0.76 | 0.007 |
| Carbohydrate N/A for diabetes; ordered eligibility-adjusted adherence | 0.49 | 0.25–0.97 | 0.041 |
| Carbohydrate removed for all patients; 13-item count | 0.72 | 0.37–1.41 | 0.337 |
| Primary 14-item count with diabetes additionally adjusted | 0.64 | 0.31–1.33 | 0.231 |

All 18 patients with diabetes were recorded as not receiving carbohydrate loading and were also contained within the chronic-comorbidity indicator. The attenuation of the comorbidity estimate when carbohydrate is excluded or diabetes is modeled separately indicates score-construction sensitivity; the primary comorbidity coefficient is therefore not treated as a robust independent implementation finding. The adherence-factor analysis is exploratory throughout.

## Supplementary Table S6. Hospital length-of-stay model comparison

| **Model** | **Log-likelihood** | **AIC** | **Dispersion / α** | **Diagnostic** |
| --- | --- | --- | --- | --- |
| Poisson | -264.77 | 555.53 | 0.5 | Converged |
| Negative binomial | -264.77 | 557.53 | 1.51e-09 | Alpha approached Poisson boundary; convergence warning possible |
| Zero-truncated Poisson | -261.44 | 548.88 |  | Converged; selected primary model |
| Zero-truncated negative binomial | -261.44 | 550.88 | 1e-08 | Dispersion collapsed toward Poisson boundary |

AIC, Akaike information criterion. The standard Poisson Pearson dispersion statistic was 0.50, indicating underdispersion relative to the ordinary Poisson variance assumption rather than overdispersion. The negative-binomial dispersion estimate collapsed to α approximately 1.0 × 10⁻8, effectively the Poisson boundary. The zero-truncated Poisson model therefore provided the lowest AIC without a dispersion-boundary problem.

**Supplementary Table S6b. Selected zero-truncated Poisson residual and influence diagnostics**

| **Diagnostic** | **Result** | **Interpretation** |
| --- | --- | --- |
| Pearson residual dispersion (Σr²/df) | 0.52 | No evidence of residual overdispersion |
| Likelihood-based deviance/df | 0.46 | No evidence of residual overdispersion |
| Maximum absolute Pearson residual | 3.68; study ID 121; LOS 14 days | Largest Pearson residual |
| Maximum absolute deviance residual | 3.04; study ID 121; LOS 14 days | Largest likelihood-based deviance residual |
| Largest absolute deletion DFBETA for adherence coefficient | 1.09; study ID 6; adherence 64.3%; LOS 19 days | Most influential observation for the adherence coefficient |
| Primary adherence estimate after deleting study ID 6 | Adjusted IRR 0.86; 95% CI 0.78–0.95; p = 0.004 | Direction persisted with attenuated magnitude |

Pearson residual dispersion was calculated as the sum of squared Pearson residuals divided by residual degrees of freedom. Likelihood-based deviance residuals were calculated from the difference between each fitted zero-truncated Poisson likelihood contribution and the corresponding saturated zero-truncated Poisson contribution. Case influence was assessed by exact leave-one-out refitting of all 145 observations; deletion DFBETA for the adherence coefficient was standardized by the primary HC1 standard error. Study ID 6, the only patient with 9 of 14 completed items (64.3% adherence), had the longest LOS of 19 days and was the dominant influence on the adherence coefficient.

## Supplementary Table S7. Sensitivity analyses for discharge-excluded ERAS adherence and hospital length of stay

| **Analysis** | **N** | **Adjusted IRR per 10 percentage points** | **95% CI** | **p-value** |
| --- | --- | --- | --- | --- |
| Primary discharge-excluded 14-item score | 145 | 0.79 | 0.66–0.93 | 0.005 |
| Exclude 5 component-defined complications | 140 | 0.86 | 0.79–0.95 | 0.002 |
| Exclude all 6 F1-positive complication records | 139 | 0.86 | 0.78–0.95 | 0.002 |
| Add 5 component-defined complication indicator | 145 | 0.83 | 0.76–0.91 | <0.001 |
| Add 6-record F1 complication indicator | 145 | 0.81 | 0.73–0.89 | <0.001 |
| Exclude LOS 19 | 144 | 0.86 | 0.78–0.95 | 0.004 |
| Exclude LOS 14 | 144 | 0.77 | 0.65–0.92 | 0.004 |
| Exclude LOS 11 | 144 | 0.80 | 0.67–0.95 | 0.011 |
| Exclude LOS 11, 14, or 19 | 142 | 0.87 | 0.79–0.96 | 0.004 |
| Carbohydrate N/A in diabetes | 145 | 0.78 | 0.67–0.91 | 0.002 |
| Carbohydrate removed from adherence score | 145 | 0.80 | 0.70–0.92 | 0.001 |

All LOS sensitivity models used the primary covariate set unless otherwise stated. Complication adjustment was considered a sensitivity analysis because postoperative complications may lie downstream of adherence. The inverse association persisted in every analysis, with adjusted IRRs ranging from 0.77 to 0.87; the estimate attenuated toward 0.86–0.87 in analyses that excluded postoperative complications or the longest hospital stays.

## Supplementary Table S8. Phase-specific adherence sensitivity analysis

| **Phase** | **Adjusted IRR per 10 percentage points** | **95% CI** | **p-value** |
| --- | --- | --- | --- |
| Preoperative | 0.91 | 0.75–1.10 | 0.328 |
| Intraoperative | 0.96 | 0.85–1.09 | 0.537 |
| Postoperative, excluding discharge | 0.93 | 0.89–0.97 | 0.001 |

Discharge by postoperative day 3 was excluded from the postoperative phase. The postoperative estimate is particularly susceptible to reverse causation because adherence and recovery were measured during the same postoperative interval.

## Supplementary Table S9. Data-quality reconciliation log

| **Issue** | **Affected deidentified study IDs** | **Resolution** |
| --- | --- | --- |
| Calendar-date format/chronology | 7,16,24,30,45,63,78,79,84,110,114,119,131,136,137,144 | Exact dates were not used to derive LOS. Dedicated LOS field F2 retained; no patient removed. |
| Age derivation | 93,94,95,96,97 | Age derived from surgery year in A10 minus year of birth A6. A2 was not used; these five records differ by one year from an A2-based derivation. |
| Hemoglobin/anemia summary discrepancy | 3,17,18,76,126 | Preoperative anemia derived from measured hemoglobin <120 g/L; inconsistent C19 summary flag not used. |
| Broad indication overlap | 15,21 | Both C1 and C3 positive. Prespecified hierarchy used C2 > C3 > Other, so these records were classified as myometrial. |
| Broad indication no positive C1-C5 field | 64 | Classified as Other benign indication. |
| Complication summary flag | 136 | F1 positive while all available component-specific complication fields were negative. Primary outcome used component-defined algorithm; sensitivity treated all six F1-positive records as complications. |
| VTE assessment flag | 125 | C28=0 but C29 contained an explicit Caprini-derived risk category. Risk assessment considered documented; prophylaxis concordance was not inferred from C29-C30. |

The oral-intake (E14) and mobilization (E24) fields are coded with 0 indicating completion within 6 hours, inverse to the polarity of the other completion fields; the resulting counts (52/145 and 20/145) reproduce the component rates of the original institutional audit and the mean of 11.66 of 15 completed items. No patient was removed because of a data-quality discrepancy. Direct identifiers, medical-record numbers, names, addresses, and exact calendar dates are not included in the analytic dataset or this supplement.

## Supplementary Table S10. Standardized expected hospital length of stay at the observed discharge-excluded ERAS completion counts

| **Completed items (of 14)** | **Observed adherence, %** | **Observed n** | **Standardized expected LOS, days (95% CI)** |
| --- | --- | --- | --- |
| 9 | 64.3 | 1 | 6.05 (4.57–8.21) |
| 10 | 71.4 | 14 | 5.12 (4.35–6.17) |
| 11 | 78.6 | 73 | 4.35 (4.07–4.73) |
| 12 | 85.7 | 46 | 3.71 (3.44–4.08) |
| 13 | 92.9 | 11 | 3.20 (2.74–3.84) |

Standardized conditional means were computed from the multivariable zero-truncated Poisson model at the five completion counts actually observed in the cohort. The corresponding adherence values were 64.3%, 71.4%, 78.6%, 85.7%, and 92.9%. Confidence intervals used 50,000 common multivariate-normal coefficient draws from the explicit HC1 covariance matrix with random seed 1907048. These are model-standardized predictions evaluated at observed exposure support values rather than raw within-group means. The estimate at 9 of 14 completed items is based on an exposure level represented by one patient and is therefore especially sensitive to leverage at the lower end of the adherence distribution.

**Supplementary Table S11. Functional-form sensitivity of the adherence–LOS association**

| **Analysis** | **N** | **Linear adherence term** | **Quadratic coefficient** | **Quadratic p-value** | **AIC** |
| --- | --- | --- | --- | --- | --- |
| Primary linear ZTP | 145 | Adjusted IRR 0.79 (95% CI 0.66–0.93); p = 0.005 | Not included | Not applicable | 548.88 |
| Centered linear + quadratic ZTP | 145 | IRR at 80% adherence 0.77 (95% CI 0.69–0.87); p < 0.001 | 0.201 (95% CI 0.039–0.363) | 0.015 | 545.14 |
| Linear ZTP excluding LOS = 19 days | 144 | Adjusted IRR 0.86 (95% CI 0.78–0.95); p = 0.004 | Not included | Not applicable | 532.51 |
| Centered linear + quadratic ZTP excluding LOS = 19 days | 144 | IRR at 80% adherence 0.86 (95% CI 0.77–0.97); p = 0.010 | 0.008 (95% CI −0.128 to 0.144) | 0.903 | 534.51 |

ZTP, zero-truncated Poisson; AIC, Akaike information criterion; IRR, incidence rate ratio; CI, confidence interval. All models used the same covariate set as the primary LOS analysis. For quadratic models, centered adherence was defined as z = (adherence percentage − 80)/10 and both z and z² were entered. The reported linear-term IRR in a quadratic model is therefore the local multiplicative slope at 80% adherence rather than a single global IRR. The full-cohort quadratic term was statistically detectable, but it became essentially null after exclusion of the single lowest-adherence patient with the 19-day stay. The linear inverse association remained directionally consistent after that exclusion. These findings support interpretation of the primary linear IRR as an average model-based association rather than evidence of a strictly linear biological dose-response.

## Supplementary statistical reproducibility statement

The analysis was reconstructed from the locked 145-patient source workbook and separated into two reproducibility layers. The internal build script (build_analytic_dataset.py) converts the protected source workbook into a deidentified analytic CSV without exporting names, medical-record numbers, addresses, occupations, or exact calendar dates. The reproducibility analysis script (run_analysis_from_deidentified_csv.py), when used with the authorized deidentified analytic CSV, recreates the primary ordinal adherence model, 1,000-replicate bootstrap stability analysis, proportional-odds sensitivity analyses, carbohydrate-score construction sensitivities, count-model comparison, primary zero-truncated Poisson model with explicit HC1 covariance, standardized LOS predictions using 50,000 coefficient draws, complication sensitivities, phase-specific analysis, selected-model residual diagnostics, exact leave-one-out influence assessment, centered quadratic functional-form sensitivity analyses, and Figure 2. The journal-facing reproducibility archive contains the analysis code, pinned software requirements, operational dictionary, checksums, and aggregated outputs; patient-level data are retained under institutional governance. Qualified researchers may request the deidentified minimal analytic dataset from the corresponding author, subject to approval by Nhan Dan Gia Dinh Hospital and applicable ethics and data-protection requirements. Random seed 1907048 is used for bootstrap and simulation-based uncertainty calculations.
